# Numerical evaluation on behavior of an integrated slope stabilization structure under seismic effect

**DOI:** 10.1038/s41598-026-47573-9

**Published:** 2026-04-05

**Authors:** Yujia Wang

**Affiliations:** School of Civil Engineering, Southwest Forestry University, Kunming, China

**Keywords:** Pseudo-static analysis, Limit analysis, Slope stabilization, Laterally loaded piles, Engineering, Natural hazards, Solid Earth sciences

## Abstract

The issue of slope instability is common in the South Gippsland area of Victoria, Australia. To improve the current slope instability circumstance, an integrated slope stabilization structure, which involves two methods, the geogrid-reinforced soil retaining wall with gabion basket facing and the laterally loaded embedded piles, has been adopted. The I-beam, which is embedded in the laterally loaded piles, is soldered to the horizontal rail to buttress the retaining structure. To evaluate the effectiveness of this integrated structure under seismic conditions, the two-dimensional finite element limit analysis approach is adopted for pseudo-static analysis of slope stability. The behavior of the gabion basket and soil is described by the Mohr-Coulomb yield criterion. With the assistance of upper and lower bounds theorems within the limit analysis method, the highest lower bound and the lowest upper bound can be obtained to narrow the range of slope stability subjected to seismic conditions. The parametric study related to geogrid embedment length and pile embedment length has been conducted to evaluate the seismic resistance of this integrated structure. The numerical result indicates that this integrated slope stabilization structure makes a considerable improvement to the seismic resistance of the slope.

## Introduction

Slope instability is a common type of geological hazard that causes severe damage to civil structures^1,2^. The risk of slope instability in South Gippsland is high because of the mountainous terrain, and the ground shaking of earthquakes makes the condition more serious. To improve slope stability and eliminate potential negative effect of slope failure, a special slope stabilization structure which places laterally loaded piles at toe of retaining structure is adopted^3^. The facing of this retaining wall is flexible, which is composed of gabion baskets, this feature makes the structure insensitive to the ground shaking from the earthquake^4^. The reinforced soil retaining wall has been widely adopted due to its economic and technical advantages, and its seismic capability has also received much attention from the literature^5–9^. A previous study^10^ conducted several shaking table tests on a bronze strip-reinforced soil retaining wall with reduced scale and indicated that placing long reinforcement layers at a higher position of the backfill soil can avoid overturning failure effectively. Another study^11^ adopted shaking table tests to investigate the influence of design parameters (i.e., vertical spacing, stiffness, and length) on the seismic performance of reinforced soil retaining walls. The experimental result demonstrated that the design parameters impose significant influences on the structure seismic response and proposed several suggestions to the seismic design method. In addition, researchers^12^ investigated the effect of geogrid which placed in saturated backfill of reinforced soil retaining wall with a rigid panel facing under seismic condition. The result from experiments indicates that the geogrid layers can decrease the seismic deformation and resist lateral deformation effectively under seismic condition.

Compared with the conventional shaking table test model, the numerical method has also been adopted to study the response of geogrid reinforced soil walls under seismic conditions^13–15^. The previous study^16^ adopted the two-dimensional dynamic finite element method with a focus on the interface behavior to study the seismic performance of geosynthetic reinforced soil segmental retaining walls. The numerical result indicates that the shear property of interfaces is important for the seismic design of reinforced soil retaining walls and demonstrates that the dynamic tensile force in geosynthetic layers is cumulative during the shaking process. Moreover, previous researchers^5^ conducted the parametric studies of material damping ratios, numerical grid, and boundary conditions to study the impact of these parameters on the finite difference method predicted seismic wall response. They found that wall displacement diminishes with the increase of reinforcement length and stiffness, but this positive effect decreases when reinforcement length is beyond wall height. Another study^17^ adopted a finite element procedure to investigate responses of two model walls under vertical and horizontal seismic loadings, and numerical results demonstrated that vertical seismic loading increases the load in reinforcements but reduces residual facing displacements.

As another well-developed slope-stabilization structure, the seismic capability of the anchored pile structure has also been investigated thoroughly. The previous researchers^18^ conducted a seismic analysis of a single pile considering linear behavior of soil based on the boundary element models. Another study^19^ adopted the pseudo-static method to evaluate the horizontal displacements and maximum internal forces of the pile. The previous study^20^ conducted a dynamic centrifuge test on a soil slope model stabilized by an anchored pile structure to study the seismic response of the slope and piles. The result indicates that the pile is the major load-bearing structure, and failure of the slope under the seismic loading is mainly caused by slope displacement and pile bending. The researcher^21^ carried out the shaking table test to study the seismic response of the soil slope stabilized by pile groups. The experimental result demonstrates that anchored pile structures can stabilize the slope effectively under strong seismic loading conditions, and some reasonable measurements are proposed that can enhance the seismic response further. In addition, another study^22^ conducted a series of reduced-scale shaking table tests to investigate the soil arching effect of pile-stabilized soil slope subjected to seismic loading. The test result indicates that the seismic performance of piles and soil arching load-transfer ability depends on soil density and pile spacing, and a wider spacing of the pile group enhances the soil arching load-transfer ability.

Based on the literature review, both the geogrid-reinforced soil retaining wall and laterally loaded pile-stabilized slope have great seismic capability. For the slope stabilization structure adopted in the South Gippsland area, which integrates two aforementioned structures, the seismic resistance has received less attention. In addition, the knowledge of this integrated structure under seismic loading conditions can provide several critical insights for a reasonable design. In this study, the pseudo-static method is adopted to study the seismic resistance of this integrated slope-stabilization structure. A series of parametric studies regarding the geogrid embedment length and the pile embedment length are conducted. The result from the parametric study indicates that this integrated slope stabilization structure can improve the seismic capability of slopes effectively. Moreover, the responses of slopes and behaviors of the geogrid and pile under the seismic effect are also investigated.

## Study area

### Background

**Fig. 1:**
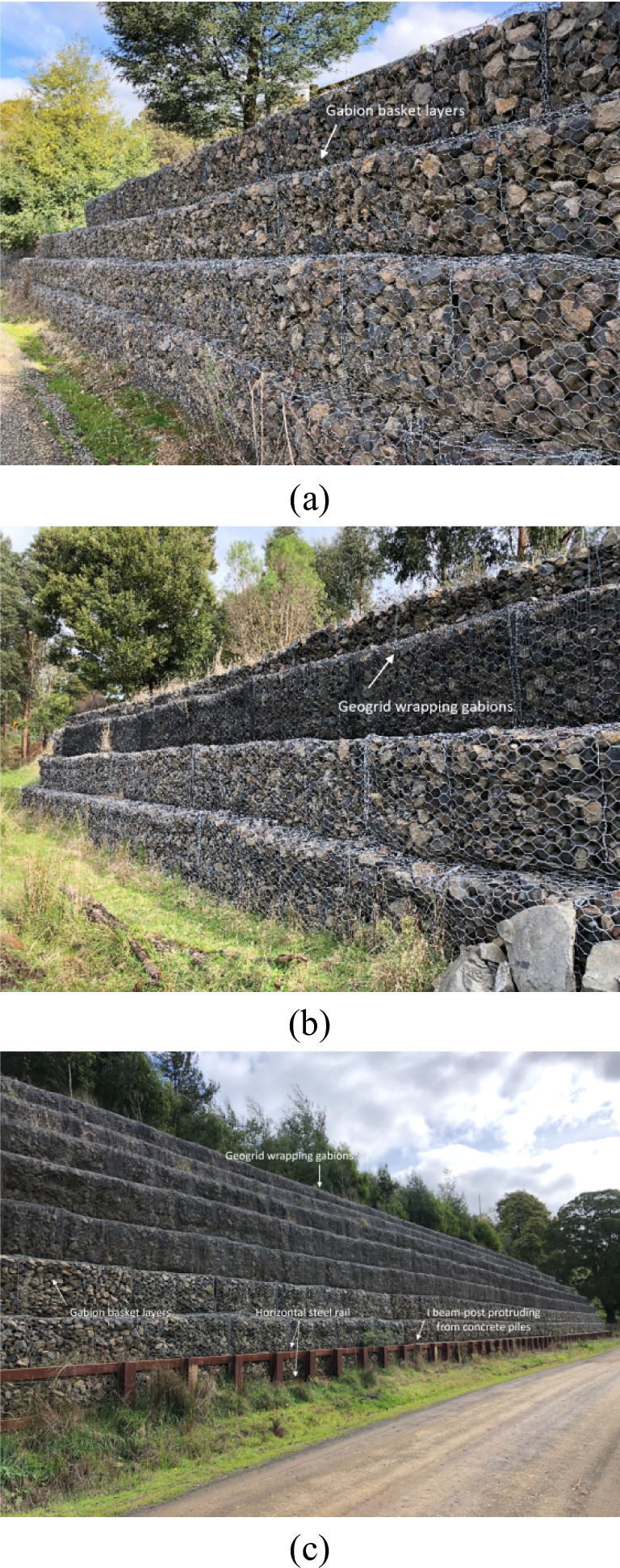
Study area background: (**a**) Gabion basket retaining wall; (**b**) Geogrid reinforced soil retaining wall; (**c**) Integrated slope-stabilization structure^25^.

The Gippsland area is in Victoria state which located in south-eastern of Australia. For agricultural purposes in 1800s, 90% of the woodland once covered Gippsland area has been cleared, which causes serious soil erosion and imposes negative effect on soil slope stability^23^. Moreover, the Gippsland area is a seismically active area within Australia and has significant earthquake vulnerability, characterized by ongoing northwest-to-southeast compression that causes frequent low-magnitude earthquake and periodic significant events^24^. The Latrobe Valley is one of the highest risk zones for ground shaking in Australia due to the ongoing tectonic pressure forces the crust to buckle. This region experiences a high density of annual minor earthquakes, with a historical frequency of significant magnitude $\:4.0$ to $\:6.0$ earthquake events roughly every three years. To enhance the slope stability within this area, retaining structures with flexible facing of gabion baskets and geogrid-reinforced soil retaining walls have been adopted for soil slopes with gentle gradients and small scale (Fig. 1a, b). To stabilize large-scale roadside embankments and treat landslides, laterally loaded pile groups are placed at toe of reinforced soil retaining walls to form an integrated structure (Fig. 1c). It is worth noting that a continuous buttress which is composed of a steel rail and I-beam presses against reinforced soil retaining wall to provide lateral support.

### Construction process

**Fig. 2:**
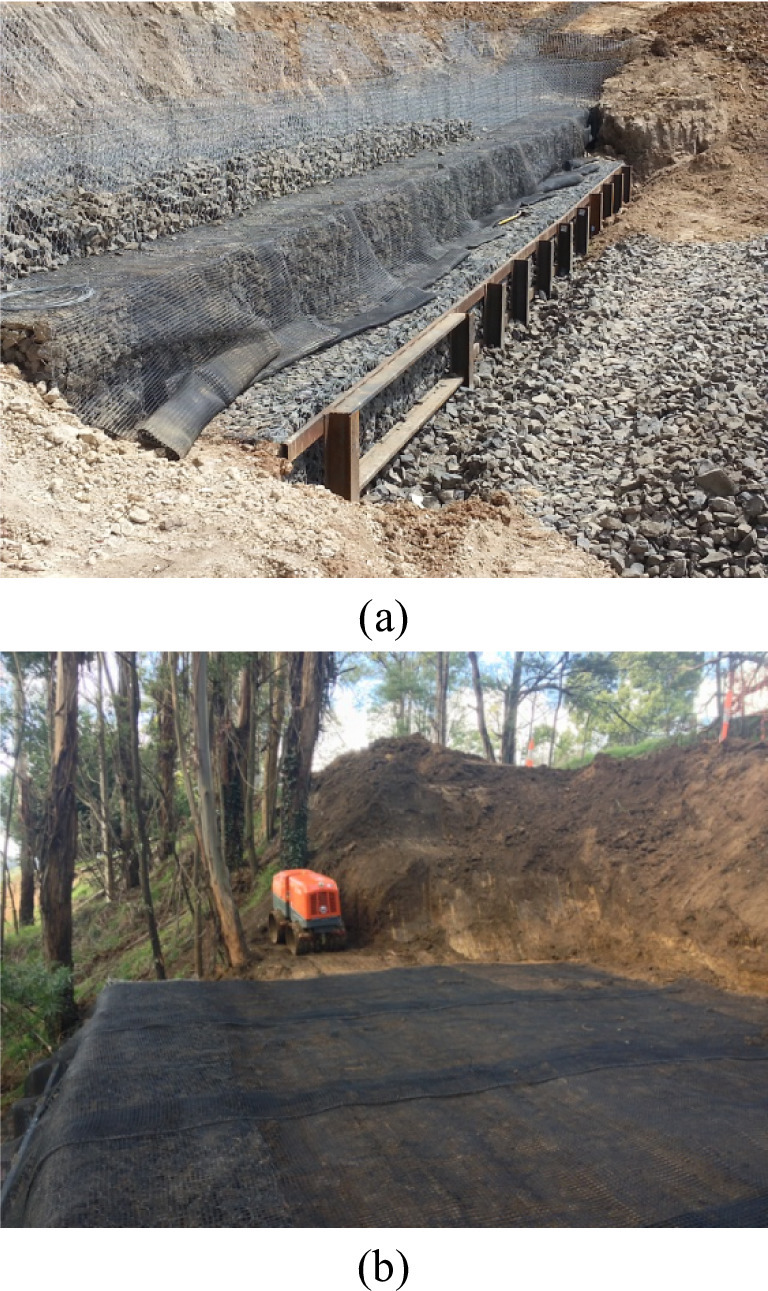
Construction process: (**a**) Pile placement prior to retaining wall; (**b**) Compaction process.

The laterally loaded piles are placed before the construction of reinforced soil retaining walls (Fig. 2a). The spiral drill is used to drill boreholes in which steel I-beams are placed and temporarily sustained when the borehole is backfilled with concrete. The steel I-beam post is 1 m longer than the drilled boreholes, and this part is then soldered to a continuous horizontal rail to brace reinforced soil retaining walls. The reinforced soil retaining wall is then placed adjacent to laterally loaded piles. The wall facing is composed of gabion basket layers, and each layer is wrapped by geogrid which is embedded into a compacted soil zone (Fig. 2b).

### Slope configurations

**Fig. 3:**
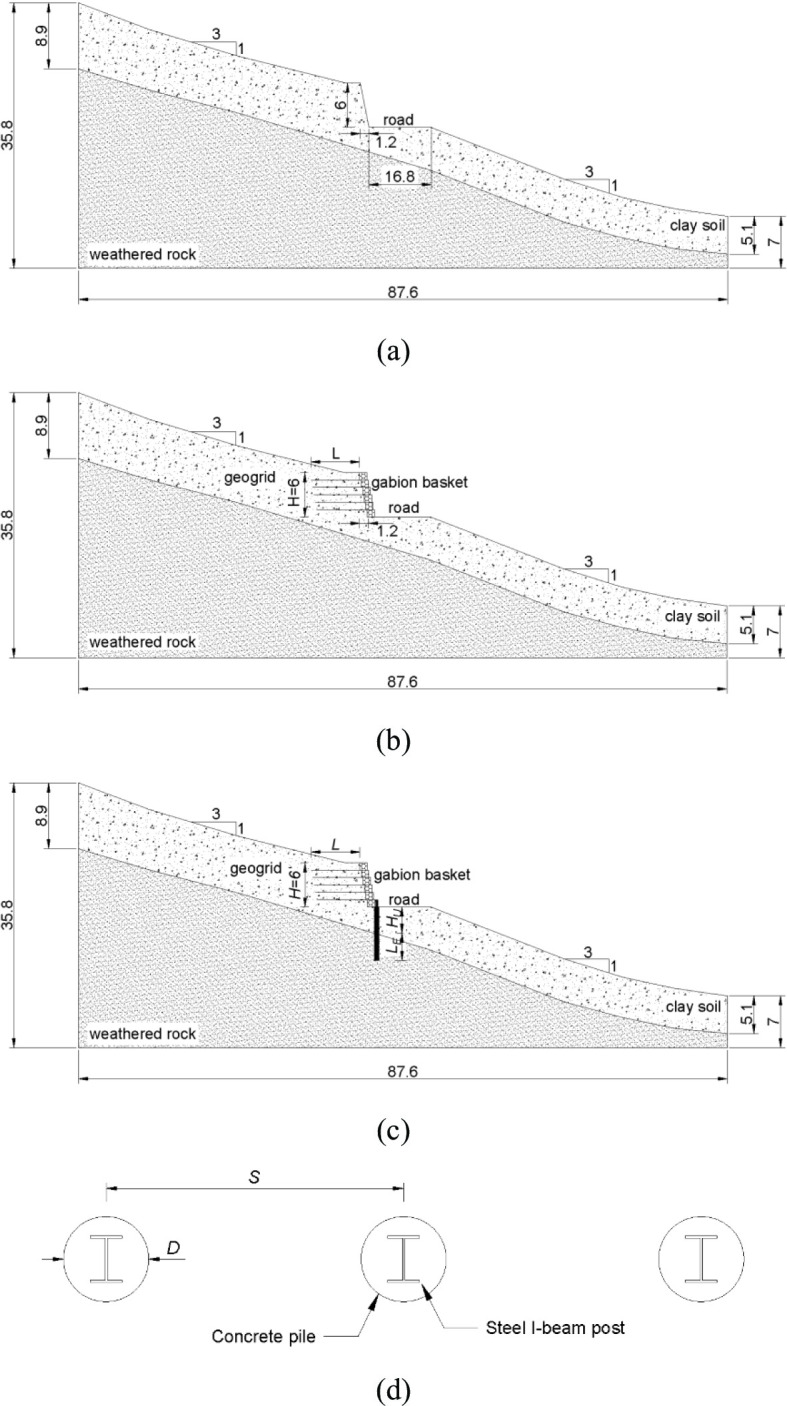
Three slope configurations: (**a**) Unreinforced road cutting; (**b**) Reinforced soil retaining wall; (**c**) Integrated structure reinforced slope; (**d**) *S* to *D* ratio.

To evaluate the response of the integrated slope stabilization structure under seismic effects, three typical slope configurations according to the site condition are adopted for numerical analysis, which are completely consistent with the actual slope project in the South Gippsland area. The essential geometric dimensions of the slope configurations analyzed in current study, including the total height of the road embankment, the thickness of the upper unstable clay soil layer, the thickness of the underlying stable weathered rock layer, as well as the slope gradient, are all derived from the field geological survey data of the study area conducted by South Gippsland Shire Council Engineering Department. The first typical slope pattern is the natural slope which is crossed by the road cutting without stabilization measurements (Fig. 3a). It can be seen that the natural slope configuration has a gradient of $\:1V:3H$. The thickness of the unstable clay soil layer is $\:8.9m$ and $\:5.1m$ at the crest and the toe of the slope, respectively. The second representative slope pattern is the geogrid-reinforced soil retaining wall (Fig. 3b). It is noticeable that the total height of the road embankment is $\:6m$ with a gradient of $\:5V:1H$. Regarding third typical slope pattern, the roading cutting is reinforced by aforementioned integrated slope stabilization structure (Fig. 3c). According to Fig. 3c, the unstable soil layer height at the toe of the road embankment in which the piles are placed is $\:3.6m$. The graphical illustration of ratio of spacing (*S*) of pile group to diameter (*D*) of single pile is also depicted in Fig. 3d. For second slope configuration, a parametric study related to ratio of length ($\:L$) of geogrid layers to the height ($\:H$) of embankment is carried out. The adopted length of geogrid layers and the corresponding $\:L/H$ ratio are listed in Table 1. Regarding the third slope pattern, another parametric study related to the ratio of pile embedded length in rock layer ($\:{L}_{E}$) to the unstable soil layer height ($\:{H}_{U}$) is conducted. The pile embedded length and the corresponding $\:{L}_{E}/{H}_{U}$ ratio are summarized in Table 2.

**Table 1:** The Value of Geogrid Embedment Ratio ($\:L/H$).

Case No.	Geogrid length $\:L$ ($\:m$)	Slope height $\:H$ ($\:m$)	$\:L/H$
1	$\:0.6$	$\:6$	$\:0.1$
2	$\:1.8$	$\:6$	$\:0.3$
3	$\:3$	$\:6$	$\:0.5$
4	$\:4.2$	$\:6$	$\:0.7$
5	$\:5.4$	$\:6$	$\:0.9$
6	$\:6.6$	$\:6$	$\:1.1$
7	$\:7.8$	$\:6$	$\:1.3$
8	$\:9$	$\:6$	$\:1.5$

**Table 2:** The Value of Pile Embedment Ratio ($\:{L}_{E}/{H}_{U}$).

Case No.	Embedded pile $\:{L}_{E}$ ($\:m$)	Unstable layer height $\:{H}_{U}$ ($\:m$)	$\:{L}_{E}/{H}_{U}$
1	$\:0$	$\:3.6$	$\:0$
2	$\:1.2$	$\:3.6$	$\:1/3$
3	$\:2.4$	$\:3.6$	$\:2/3$
4	$\:3.6$	$\:3.6$	$\:1$
5	$\:4.8$	$\:3.6$	$\:4/3$
6	$\:6$	$\:3.6$	$\:5/3$
7	$\:7.2$	$\:3.6$	$\:2$

## Theoretical considerations

### Pseudo-static approach

The pseudo-static method has been used broadly for the assessment of the slope stability under seismic loading. Regarding the pseudo-static approach, the earthquake-induced dynamic loadings are represented by body acceleration, which is imposed statically. In the pseudo-static approach, the influence of time history of the slope response to the ground shaking and other deformation-dependent or time-dependent factors (i.e., excess pore pressure generation) is neglected in this approach. The principle of current method is that earthquake-induced dynamic loads are simplified into a constant horizontal inertial body force uniformly applied to the slope, which is imposed in a static analysis framework. For pseudo-static approach, the vertical acceleration is kept fixed at $\:{g}_{v}=9.81\:m/{s}^{2}$ while the horizontal acceleration is increased incrementally until the slope reaches the limit equilibrium state of the failure. The critical seismic coefficient, $\:{k}_{c}$, which is the ratio of seismic acceleration to the gravity acceleration $\:g$ ($\:=9.81\:m/{s}^{2}$), is adopted to indicate the slope seismic stability, and can be given by:

1 $$\:{k}_{c}={g}_{h}/{g}_{v}$$

where $\:{g}_{h}$ is the horizontal seismic acceleration, $\:{g}_{v}$ is the default downward gravitational acceleration, and $\:{k}_{c}$ is the critical seismic coefficient yielding the factor of safety of slopes equals unity. In practical engineering, the critical seismic coefficient is generally taken as $\:0.4$ which covers the maximum ground motion level of historical earthquakes in the study area, and balances structural safety and economic rationality in practical engineering design. It is worth noting that in current study, the value of critical seismic coefficient is extended to $\:0.5$ to evaluate the ultimate seismic performance of the integrated slope stabilization structure under rare earthquake conditions, and to completely capture the evolution law of slope stability from serviceable state to the failure state.

### Limit analysis method

The Limit analysis method uses theorems of upper and lower bound of theory of plasticity to determine rigorous solutions of upper and lower bound for stability problems. For lower bound theory, the loads supported by the stress field that are statically admissible are lower than actual collapse loads, and a statically admissible stress field satisfies equilibrium equations, stress boundary conditions, and the yield criterion of the soil mass. Regarding upper bound theory, the loads supported by the field of velocity, which is kinematically admissible, are greater than the true collapse load, and a kinematically admissible velocity field satisfies velocity boundary conditions, compatibility, and the flow rule of material. It is worth noting that a complicated trial-and-error procedure is requested for the search of the optimal stress and velocity fields, therefore, the finite element discretization is adopted for limit analysis approach in order to establish stress and velocity fields and then to determine rigorous upper and lower bound solutions^26,27^. In addition, the solution from the lower bound theorem and the upper bound theorem will be averaged to guarantee the simulation result is correct and accurate in the current study. In the current study, both lower bound and upper bound Finite Element Limit Analysis (FELA) are performed for each slope configuration. Regarding both lower and upper bound analysis, the Factor of Safety (FoS) of each slope configuration is taken as the target parameter for the optimization. In addition, the arithmetic mean value of the converged lower bound and upper bound solutions is adopted as the final simulation result, to guarantee the correctness and accuracy of the slope seismic performance.

### Strength reduction method

The FoS determined by the shear strength reduction method (SRM) can be adopted to demonstrate the stability of the slope under both static and seismic conditions. Regarding the SRM, the principle is to progressively and uniformly reduce the effective shear strength parameters including the effective friction angle ($\:{\phi\:}^{{\prime\:}}$) and the effective cohesion ($\:{c}^{{\prime\:}}$) until the slope reaches the limit equilibrium failure state^28^. The criterion for global slope failure is defined as the numerical non-convergence of the calculation, which is induced by the rapid displacement increase at a specified characteristic node within the computational mesh^29^. The relationship between the original shear strength and the reduced shear strength can be depicted as:

2 $$\:FoS=\frac{{c}^{{\prime\:}}}{{c}_{red}}=\frac{\mathrm{t}\mathrm{a}\mathrm{n}{\phi\:}^{{\prime\:}}}{{\left(\mathrm{t}\mathrm{a}\mathrm{n}{\phi\:}^{{\prime\:}}\right)}_{red}}$$

where $\:{c}^{{\prime\:}}$ and $\:{\phi\:}^{{\prime\:}}$ denotes the effective cohesion and effective friction angle of the soil, respectively; $\:{c}_{red}$ and $\:{\left(\mathrm{t}\mathrm{a}\mathrm{n}{\phi\:}^{{\prime\:}}\right)}_{red}$ represents the reduced shear strength of soil when the slope reaches the critical failure state, respectively.

## Numerical setup

### Constitutive model and material property

The numerical model of various slope configurations applied in the current study is analyzed by a professional software for geotechnical analysis, *OptumG2*, which uses the Adaptive Finite Element Method (AFEM) ^30^. The beam element ‘*Plate*’, which has stiffness and strength properties, is applied to represent the elastoplastic behavior of the continuous buttress which is placed at the toe of reinforced soil retaining wall. The standard beam element is adopted to represent the laterally loaded pile behavior with the consideration of the ratio of *S* to *D*. The truss elements ‘*Geogrids*’ with strength and stiffness definitions are adopted to represent the behavior of geogrids. It is worth noting that truss elements can sustain tensile forces but cannot resist bending non sustain uniaxial compression. The behavior of the soil and the gabion basket is described by the Mohr-Coulomb failure criterion, which can be given by:

3 $$\:F=\left|{\sigma\:}_{1}-{\sigma\:}_{3}\right|+\left({\sigma\:}_{1}+{\sigma\:}_{3}\right)\mathrm{s}\mathrm{i}\mathrm{n}{\phi\:}^{{\prime\:}}-2{c}^{{\prime\:}}\mathrm{c}\mathrm{o}\mathrm{s}{\phi\:}^{{\prime\:}}$$

where $\:{\sigma\:}_{1}$ is the major principal stress, and $\:{\sigma\:}_{3}$ is the minor principal stress. The Mohr-Coulomb constitutive model is adopted to characterize the mechanical behavior of gabion baskets and soil in this study, it can directly capture the shear-dominated failure mechanism that is highly consistent with the mechanical response characteristics of gabion structures. Meanwhile, the two core strength parameters of this model, cohesion and friction angle, can effectively quantify the coupling effect of rock particle interlocking and steel wire mesh confinement, making this constitutive model suitable for describing the mechanical behavior of gabion baskets in the numerical simulation.

According to the main feature of materials, the strength property is summarized in Table 3. Regarding the lower weathered rock layer and the upper clay soil layer respectively, the strength parameters are calibrated to match the typical local geotechnical conditions documented in prior studies^31,32^. The strength property of geogrid layers is also summarized in Table 4. It is worth noting that the type of geogrid is polyester (PET) geogrid (GGW) with a tensile strength of $\:45kN/m$ according to the references from the South Gippsland Shire Council. In addition, researchers^33^ conducted a series of unconfined compression tests and direct shear tests on multiple groups of gabion baskets. For the unconfined compression tests, the loading process was terminated once the steel wires of the gabion baskets fractured. In the direct shear tests, four levels of axial pressure ($\:25\:kN$, $\:50\:kN$, $\:75\:kN$, and $\:125\:kN$) were applied, with the confining pressure maintained at a constant value throughout the shearing process. Based on linear regression analysis of the test results, the shear strength parameters of the gabion baskets were derived: the effective friction angle ($\:{\phi\:}^{{\prime\:}}$) is $\:{45}^{^\circ\:}$ and the effective cohesion ($\:{c}^{{\prime\:}}$) is $\:560kPa$. It is also noteworthy that the gabion baskets still maintain residual shear strength even after the failure of the steel wire mesh.

**Table 3:** Solid Materials Properties.

Materials	Friction angle $\:{\phi\:}^{{\prime\:}}\:(^\circ\:)$	Cohesion $\:{c}^{{\prime\:}}$ ($\:kPa$)	Poisson’s ratio $\:\nu\:$	Young’s modulus $\:E$ ($\:MPa$)	Unit weight $\:\gamma\:\:(kN/{m}^{3}$)
Steel rail	-	-	$\:0.27$	$\:\mathrm{290,000}$	$\:79$
Concrete pile	-	-	$\:0.21$	$\:\mathrm{30,000}$	$\:23$
Gabion basket	$\:45^\circ\:$	$\:560$	$\:0.2$	$\:20$	$\:17$
Weathered rock	$\:36^\circ\:$	$\:180$	$\:0.3$	$\:220$	$\:22$
Clay soil	$\:30^\circ\:$	$\:30$	$\:0.23$	$\:200$	$\:20$

Note: Concrete strength C30, steel strength S500.

**Table 4:** Geogrid Properties.

Parameters	Geogrid
Structural element type	Truss
Constitutive model	Elastic-perfectly plastic model
Thickness ($\:mm$)	$\:2.5$
Elastic modulus ($\:Mpa$)	$\:\mathrm{2,600}$
Tensile strength ($\:kN/m$)	$\:45$
Tensile stiffness ($\:kN/m$)	$\:\mathrm{6,200}$
Tensile failure strain ($\:\%$)	$\:10$

### Interaction properties and boundary conditions

The current numerical study involves three types of interaction: (a) interaction between soil and gabion basket; (b) interaction between geogrid and gabion basket; (c) interaction between soil and geogrid. The interaction property is described by the Mohr-Coulomb yield criterion, and the shear stress within the interface can be depicted as:

4 $$\:{\tau\:}_{max}={c}_{int}^{{\prime\:}}+{\sigma\:}_{n}^{{\prime\:}}\mathrm{t}\mathrm{a}\mathrm{n}{\phi\:}_{int}^{{\prime\:}}$$

where $\:{\phi\:}_{int}^{{\prime\:}}={\mathrm{t}\mathrm{a}\mathrm{n}}^{-1}\left({c}_{rf}\mathrm{t}\mathrm{a}\mathrm{n}{\phi\:}^{{\prime\:}}\right)$ is interface friction angle, and $\:{c}_{rf}$ represents the reduction factor; $\:{\sigma\:}_{n}^{{\prime\:}}$ is effective normal stress; and $\:{c}_{int}^{{\prime\:}}$ represents the interface cohesion. For geogrid involved interfaces, the $\:{c}_{rf}$ is determined as $\:0.84$ to calculate the interface friction angle between the neighboring materials which is consistent with^34^. The value of $\:{c}_{int}^{{\prime\:}}$ is determined as $\:4kPa$ according to the interface properties involved in^35^. The dilation angle and the cohesion of the interface between the soil and the layers of geogrid are assumed as zero^34^. Regarding soil-gabion basket interfaces, the interaction elements are used to represent the shear strength and the stiffness between the gabion basket unit and the reinforced clay soil, the cohesion between the interface $\:{c}_{int}^{{\prime\:}}$ is adopted as $\:4kPa$, and the $\:{\phi\:}_{int}^{{\prime\:}}$ can be depicted as:

5 $$\:{\phi\:}_{int}^{{\prime\:}}={\mathrm{t}\mathrm{a}\mathrm{n}}^{-1}(0.8\mathrm{t}\mathrm{a}\mathrm{n}({\phi\:}_{min}^{{\prime\:}}\left)\right)$$

where $\:{\phi\:}_{min}^{{\prime\:}}$ represents minimum friction angle of the neighboring material^35^. The interaction properties are summarized in Table 5.

Regarding the displacement boundary conditions adopted in the numerical simulation, the displacement of the bottom plane of all slope configurations involved in this study is fixed in all degrees of freedom. Regarding the lateral boundaries that are perpendicular to the model base, a normal-direction constraint is applied, which restricts the horizontal displacement of the numerical model.

**Table 5:** Interaction Properties.

Parameters	Gabion-soil	Geogrid-gabion	Geogrid-soil
Cohesion ($\:{c}_{int}^{{\prime\:}}$) ($\:kPa$)	$\:4$	$\:4$	$\:4$
Friction angle ($\:{\phi\:}_{int}^{{\prime\:}}$) ($\:^\circ\:$)	$\:16^\circ\:$	$\:40^\circ\:$	$\:17^\circ\:$

### Validation

**Fig. 4:**
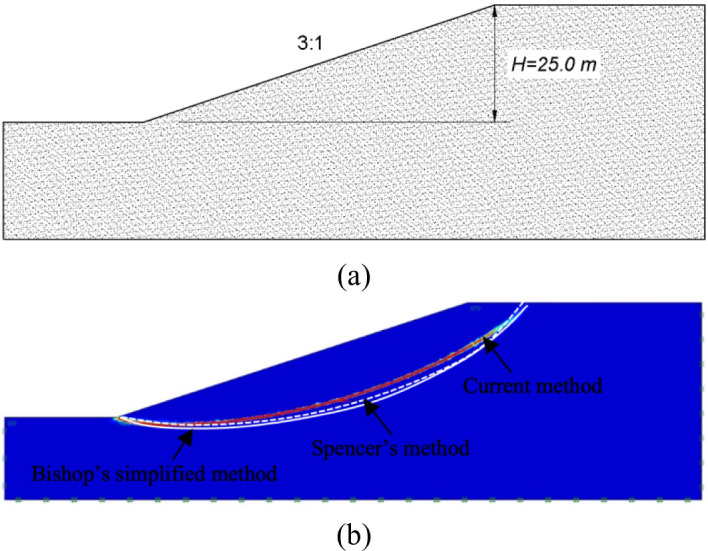
Comparison of critical slip surface: (**a**) Geometry of example slope; (**b**) Critical slip surface from current method and Loukidis et al. (2003)

The objective of this section is to validate the accuracy and reliability of the current method that employed in *OptumG2* in predicting two essential slope seismic capability indicators: the distribution of the critical slip surface and the critical seismic coefficient ($\:{k}_{c}$). A classic problem of slopes under seismic conditions which was previously analyzed by^36^, is reproduced by the current numerical approach to ensure its validity. The slope geometry is depicted in Fig. 4a: the height of the slope is $\:25.0m$ and the gradient of the slope is $\:1V:3H$ ($\:H$, horizontal; $\:V$, vertical). The material property of the slope example is summarized in Table 6. The comparison of horizontal critical seismic coefficient, $\:{k}_{c}$, between the current approach and other methods is summarized in Table 7. It is clear that the results obtained from the present method ($\:0.429-0.431$) are in great consistency with other established methods: it deviates only by $\:0.7\%$ from Bishop’s simplified method and $\:0.2\%$ from Spencer’s method, the reliability of the present method in predicting the slope seismic capability can be verified. Moreover, the critical slip surface of the example slope derived from the present method is compared with the Spencer’s method and the Bishop’s simplified method (Fig. 4b). The great agreement is observed between the shape and depth of the critical slip surface predicted by the proposed method and those derived from the established reference methods, which further validates the capacity of the present approach to capture the failure mechanisms of slopes. In general, the reliability of the proposed method for the evaluation of slope seismic performance is verified by both the quantitative comparison of critical seismic coefficients and the qualitative consistency of critical slip surface configurations.

**Table 6:** Material Properties of the Example Slope.

Material properties	Friction angle $\:{\phi\:}^{{\prime\:}}\:(^\circ\:)$	Cohesion $\:{c}^{{\prime\:}}$($\:kPa$)	Unit weight $\:\gamma\:\:(kN/{m}^{3}$)
	$\:30^\circ\:$	$\:25$	$\:20$

**Table 7:** Comparison of Critical Seismic Coefficient ($\:{k}_{c}$).

Analysis method	$\:{k}_{c}$
Numerical upper bound method	$\:0.454$
Numerical lower bound method	$\:0.423$
Finite element method	$\:0.433$
Log-spiral upper bound method	$\:0.432$
Spencer’s method	$\:0.431$
Bishop’s simplified method	$\:0.426$
Sarma’s method	$\:0.430$
**Present method**	$\:0.429-0.431$

## Analysis of results

### Seismic response of unreinforced road cutting

#### Effect on FoS

**Fig. 5:**
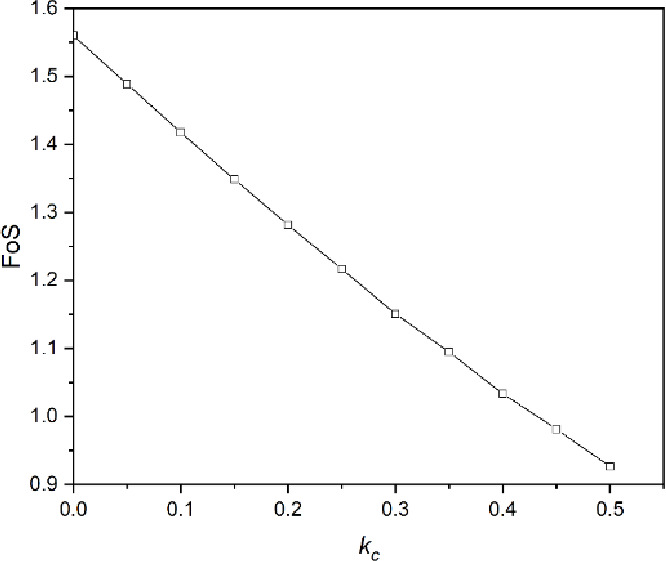
Factor of Safety (FoS) of unreinforced road cutting under seismic loading.

The FoS versus $\:{k}_{c}$ of unreinforced road cutting has been shown in Fig. 5. According to Fig. 5, the FoS of unreinforced road cutting decreases with the increase of $\:{k}_{c}$ Under static conditions ($\:{k}_{c}=0$), the FoS equals $\:1.56$ which represents a stable state. The FoS decreases to $\:0.93$ when $\:{k}_{c}$ increases to $\:0.5$ at which the slope failure takes place. It is noticeable that the relationship between FoS and $\:{k}_{c}$ of unreinforced road cutting is approximately linear.

#### Effect on critical slip surface

**Fig. 6:**
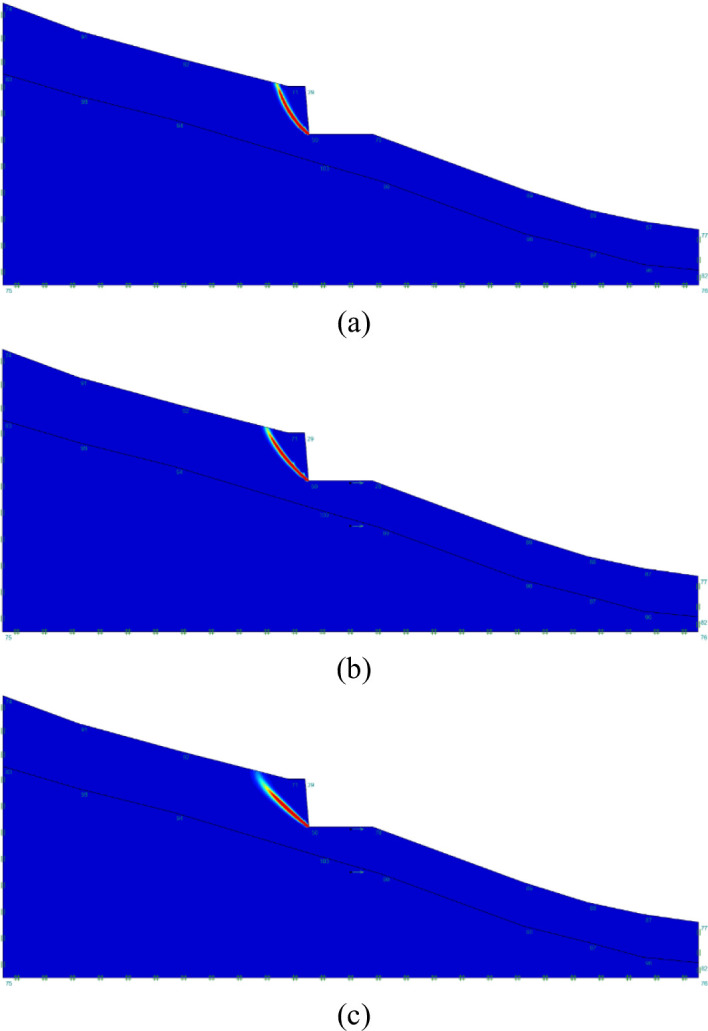
Critical slip surface of unreinforced road cutting under various values of critical seismic coefficient ($\:{k}_{c}$): (**a**) $\:{k}_{c}=0$; (**b**) $\:{k}_{c}=0.25$; (**c**) $\:{k}_{c}=0.5$

The critical sliding surface of unreinforced road cutting under various $\:{k}_{c}$ values has been presented in Fig. 6. According to Fig. 6, the failure mode is dominated by horizontal sliding and the critical sliding surface always intersects with the toe. It is notable that the width of the sliding mass increases with the increase of $\:{k}_{c}$, which accords with the phenomenon of reducing FoS.

### Seismic performance of geogrid-reinforced soil retaining wall

#### Effect on FoS

**Fig. 7:**
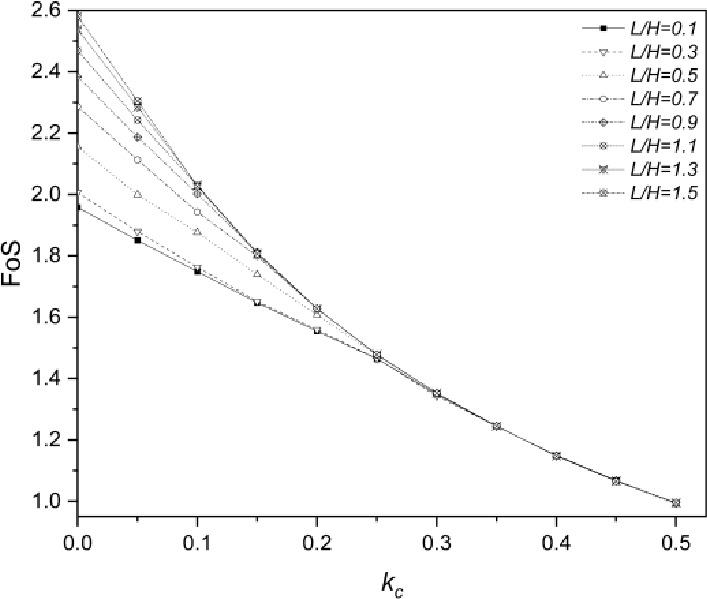
Factor of Safety (FoS) of reinforced soil retaining wall with various geogrid embedment ratios ($\:L/H$) under seismic effect.

The FoS of second slope pattern with eight $\:L/H$ ratios under seismic loading is presented in Fig. 7. Under static loading condition, the geogrid improves the stability of slopes effectively, and major improvement in FoS occurs when $\:L/H$ ratio ranges from the value of $\:0.5$ to the value of $\:1.1$. Compared with $\:L/H=0.1$ ($\:0.6m$ geogrid embedded), the percentage of the increase in FoS of slopes is around $\:32.8\%$ when $\:L/H$ increases to $\:1.5$ ($\:9m$ geogrid embedded) under static conditions. When $\:{k}_{c}$ increases to $\:0.1$, the contribution of geogrid to slope seismic capability is still significant, and the overall increase in FoS with the increase of geogrid embedment length is around $\:15.8\%$. However, the effect of geogrid on slope seismic resistance diminishes significantly regardless of various embedment lengths when $\:{k}_{c}$ grows to $\:0.2$, the improvement in FoS only reaches $\:4.8\%$. Moreover, the FoS of all configurations of geogrid-reinforced soil retaining walls is basically the same when $\:{k}_{c}$ is larger than $\:0.25$, which indicates that increasing geogrid embedment length is meaningless when the earthquake magnitude is high. The FoS of the second slope configuration with different $\:L/H$ ratios decreases to around $\:1.0$ when $\:{k}_{c}$ increases to $\:0.5$, which represents a limit equilibrium state of slopes. In addition, the nonlinear relationship between FoS and $\:{k}_{c}$ becomes more significant with the growth of the embedded geogrid length.

#### Effect on critical slip surface

**Fig. 8:**
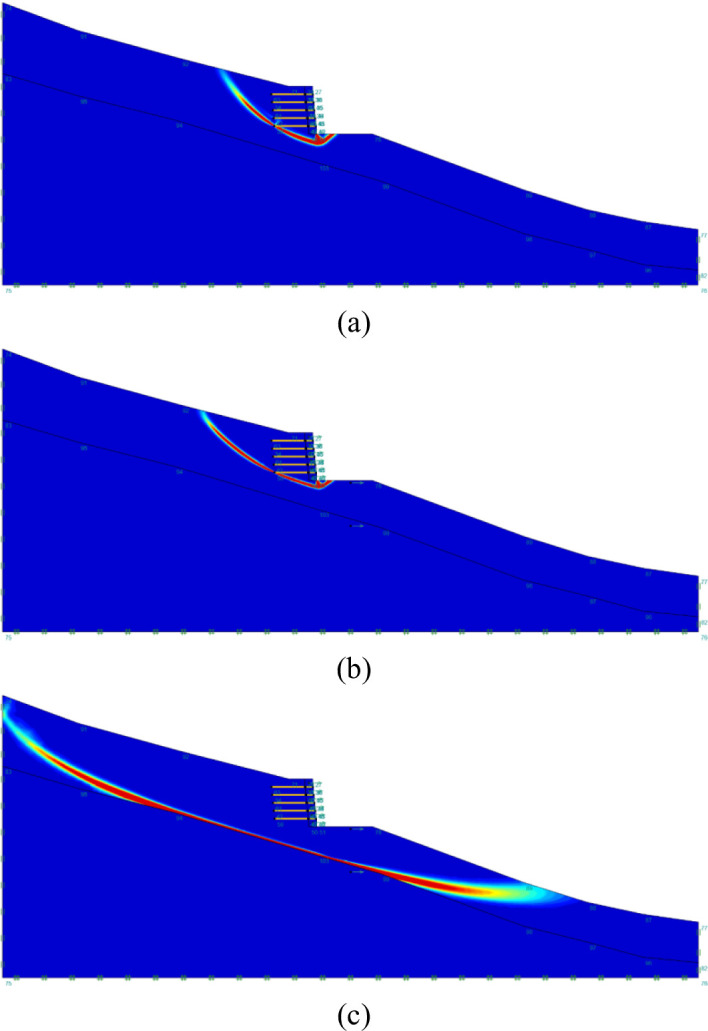
Critical slip surface of geogrid reinforced slopes with $\:L/H=0.7$ under three values of critical seismic coefficient ($\:{k}_{c}$): (**a**) $\:{k}_{c}=0$; (**b**) $\:{k}_{c}=0.15$; (**c**) $\:{k}_{c}=0.3$

The critical sliding surface of second slope configuration under three values of $\:{k}_{c}$ is depicted in Fig. 8. It is noticeable that the second slope configuration which adopts the $\:L/H$ ratio equals $\:0.7$ is selected as the failure mode of different geogrid embedment length is basically the same. It can be seen that for $\:{k}_{c}$ equals $\:0$ and $\:0.15$, the critical sliding surface beneath the toe intersects with the bottom geogrid layer at the left end, and the failure mode is dominated by horizontal sliding. When $\:{k}_{c}$ increases to $\:0.3$, the critical sliding surface extends significantly, and the failure mode can be defined as global failure. It can also be noticed that the geogrid embedment length would have a minor influence on the seismic capability of slopes when global failure mode occurs, this phenomenon can be adopted to explain the distribution of FoS when $\:{k}_{c}$ greater than $\:0.3$. It is also noticeable that the scale of the critical sliding surface extends with the growth of $\:{k}_{c}$, which is similar to the distribution of unreinforced slope failure mode.

#### Effect on tensile force of geogrid

**Fig. 9:**
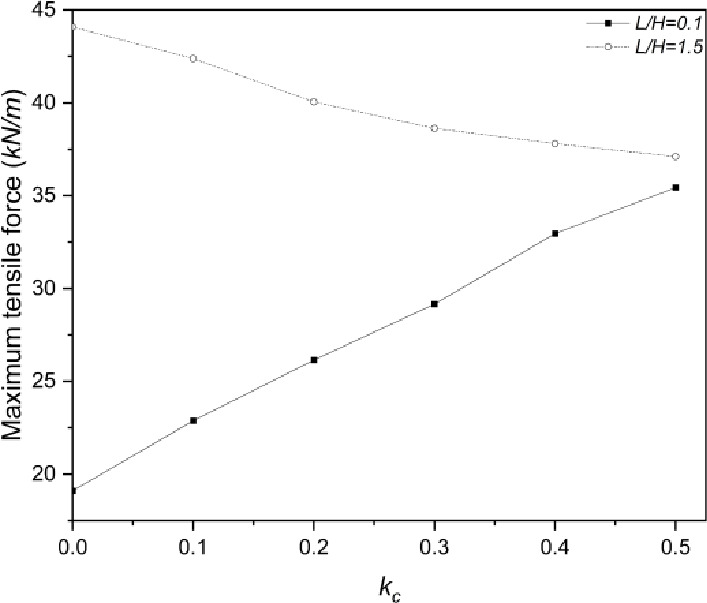
Maximum geogrid tensile force of the second slope configuration under seismic effect.

The maximum geogrid tensile force within the second slope pattern under various values of $\:{k}_{c}$ has been shown in Fig. 9. It is noticeable that the minimum and maximum $\:L/H$ ratios adopted in the parametric study are used for a clear comparison. For $\:L/H$ ratio equals $\:0.1$, the maximum tensile force within geogrid layers increases linearly from $\:19.1kN/m$ to $\:35.4kN/m$ with the increase of $\:{k}_{c}$. For $\:L/H$ ratio equals $\:1.5$, it shows a decreasing trend from $\:44.1kN/m$ to $\:37.1kN/m$. The geogrid embedment length is insufficient when $\:L/H=0.1$, therefore, the tensile strength of the geogrid is not mobilized effectively through friction force under static loading. With the growth of seismic loading, the geogrid tensile strength at the junction of soil and gabion basket is mobilized and shows an increasing trend. Regarding the $\:L/H$ ratio which is equal to $\:1.5$, the embedment length is sufficient and the tensile strength is fully mobilized under static loading circumstances. With the growth of $\:{k}_{c}$ value, the tensile property of the geogrid cannot be mobilized further and shows a decreasing trend.

The variation of geogrid tensile force in Fig. 9 directly indicates the mechanical mechanism of geogrid inhibiting shallow slope sliding and the mobilization process of its anti-sliding resistance under seismic loading. The geogrid with insufficient embedment length ($\:L/H=0.1$) cannot pass through the potential shallow slip surface, therefore its tensile strength is gradually mobilized with the increase of seismic intensity to constrain the lateral deformation of shallow soil, showing a trend of increasing tensile force. The geogrid with sufficient embedment length ($\:L/H=1.5$) fully passes through the shallow slip surface, forms a stable reinforced soil composite with the backfill soil under static conditions, and fundamentally inhibits the initiation and development of shallow sliding, therefore the required tensile force decreases slightly with the increase of seismic intensity. This phenomenon clarifies that the geogrid restricts the development of shallow slip surface through the tensile force transmitted by the soil-geogrid interface, thus realizing the effective control of shallow slope failure.

### Seismic response of integrated slope stabilization structure

#### Effect on FoS

**Fig. 10:**
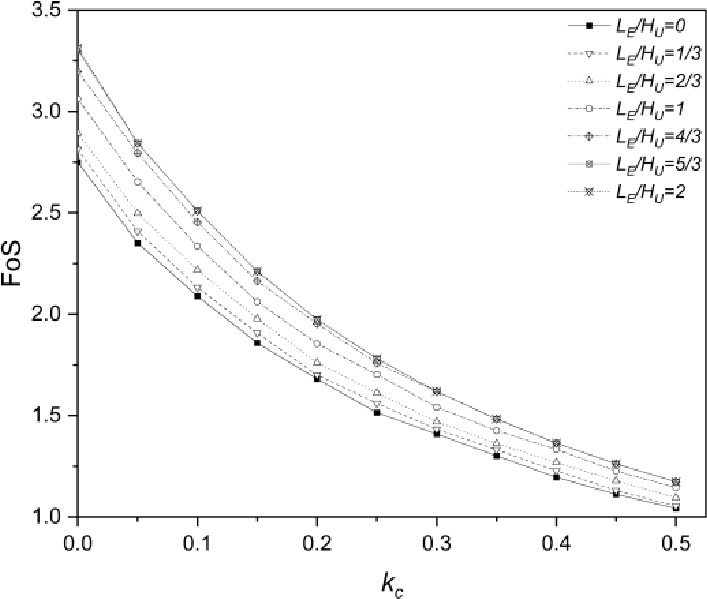
Factor of Safety (FoS) of the Integrated structure stabilized slope with various pile embedment ratios ($\:{L}_{E}/{H}_{U}$) under seismic effect.

The FoS of the integrated structure stabilized slope with seven $\:{L}_{E}/{H}_{U}$ ratios is shown in Fig. 10. It is noticeable that the geogrid embedment length involved in the third slope configuration is based on the actual site condition, which is $\:6.6m$. According to Fig. 10, the FoS decreases with the growth of $\:{k}_{c}$. Under static loading conditions ($\:{k}_{c}=0$), the FoS of slopes increases from $\:2.75$ to $\:3.31$ when $\:{L}_{E}/{H}_{U}$ ratio increases from $\:0$ to $\:2$, and the percentage is estimated at $\:20.4\%$. When $\:{k}_{c}$ increases to $\:0.5$, the FoS of slope drops significantly: $\:1.04$ for $\:{L}_{E}/{H}_{U}$ equals $\:0$ and $\:1.17$ for $\:{L}_{E}/{H}_{U}$ equals $\:2$, the percentage of increment is around $\:12.5\%$. It is clear that the larger length of pile embedment can keep slopes still at a stable state under seismic loading conditions with high magnitude. However, it is also worth to note that for all $\:{k}_{c}$ values, the major improvement in FoS occurs when $\:{L}_{E}/{H}_{U}$ increases from $\:2/3$ to $\:5/3$, and further increment in pile embedment length from $\:5/3$ to $\:2$ has negligible impact on the stability of the slope, which is similar to the effect of geogrid embedment length of second slope configuration.

#### Effect on critical slip surface

**Fig. 11:**
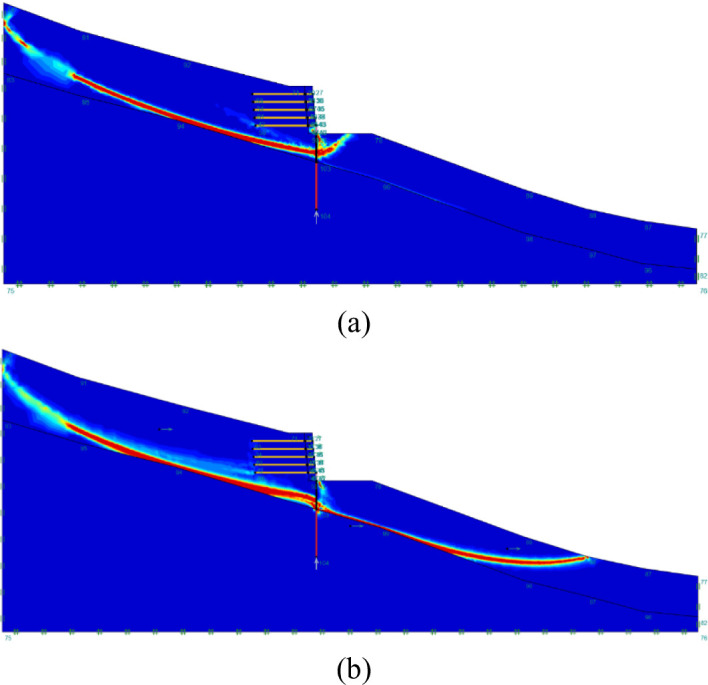
Critical slip surface of integrated structure stabilized slopes with $\:{L}_{E}/{H}_{U}=5/3$ under two values of critical seismic coefficient ($\:{k}_{c}$): (**a**) $\:{k}_{c}=0$; (**b**) $\:{k}_{c}=0.5$

The critical sliding surface of the integrated structure stabilized slope under two values of $\:{k}_{c}$ is shown in Fig. 11. The $\:{L}_{E}/{H}_{U}$ value adopted here is $\:5/3$ to indicate the failure mode of the actual site condition. It can be seen that when the loading condition is static ($\:{k}_{c}=0$), the critical slip surface that is beneath the toe of the road embankment and passing through the pile group mainly exists in the area behind the integrated structure. The failure mechanism of the slope is determined as horizontal sliding. For $\:{k}_{c}$ equals $\:0.5$, the critical sliding surface altered significantly. The right end of the critical sliding surface extends to the lower part of the entire slope, and the sliding surface length almost doubled. The failure mode can then be determined as the global failure. The change of failure mode of slopes induced by the shaking of the earthquake can be adopted to explain the phenomenon of significant decrease in FoS with the increase of $\:{k}_{c}$.

#### Effect on pile bending moment

**Fig. 12:**
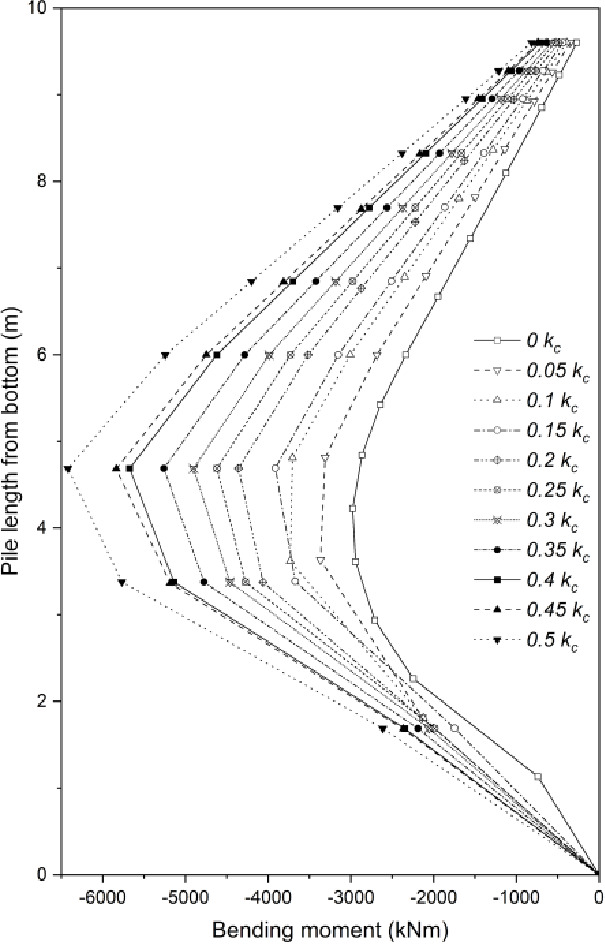
Pile bending moment of the integrated structure stabilized slope under seismic effect.

The bending moment of laterally loaded pile within third slope configuration under different values of $\:{k}_{c}$ has been depicted in Fig. 12. It is noticeable that pile length is $\:9.6m$ as the $\:{L}_{E}/{H}_{U}$ value of $\:5/3$ is adopted for the analysis. It is clear that the bending moment of the pile at the critical state increases with the growth of $\:{k}_{c}$ value, and the maximum bending moment of the pile is basically doubled when $\:{k}_{c}$ increases from $\:0$ to $\:0.5$. The interface of the rock layer and the unstable soil layer is at a distance of $\:6m$ from the pile bottom, however, the maximum pile bending moment did not occur at this position. For the lower value of $\:{k}_{c}$ ($\:0-0.1\:{k}_{c}$), the maximum pile bending moment occurs at a position where at a distance of about $\:2m$ below the interface. Regarding larger value of $\:{k}_{c}$ ($\:0.15-0.5\:{k}_{c}$), the maximum bending moment of the pile happens at the position where has a distance of around $\:1.3m$ below the unstable soil layer. It is worth noting that the position of maximum bending moment has a tendency to move upward with the increase of $\:{k}_{c}$. In general, more slide resistance of the pile is mobilized with the increase of $\:{k}_{c}$ to stabilize slopes though the FoS of slopes has a tendency to decrease.

The bending moment of the pile increases significantly with the increase of $\:{k}_{c}$, which represents that the pile mobilizes its high bending stiffness to resist the increasing sliding force from unstable soil mass under seismic loading. The upward migration of the maximum bending moment position with the increase of seismic intensity is consistent with the expansion of the slope critical slip surface and the transition of failure mode to global instability: under low seismic intensity, the pile mainly relies on the anchoring effect of the deep embedded section to resist sliding forces; with the increase of seismic intensity, the pile mobilizes the bending resistance of the full pile length to prevent the global failure of the slope. Therefore, the load transfer mechanism of the embedded pile to resist deep slope sliding through its bending performance can be demonstrated.

## Discussion

The response of three slope configurations to seismic loading conditions has been demonstrated through the finite element limit analysis method, and the behavior of geogrid and laterally loaded pile under seismic effect has also been investigated by the parametric study. In this section, the comparison of FoS among three slope configurations will be conducted to demonstrate the effectiveness of the integrated structure under seismic effects.

**Fig. 13:**
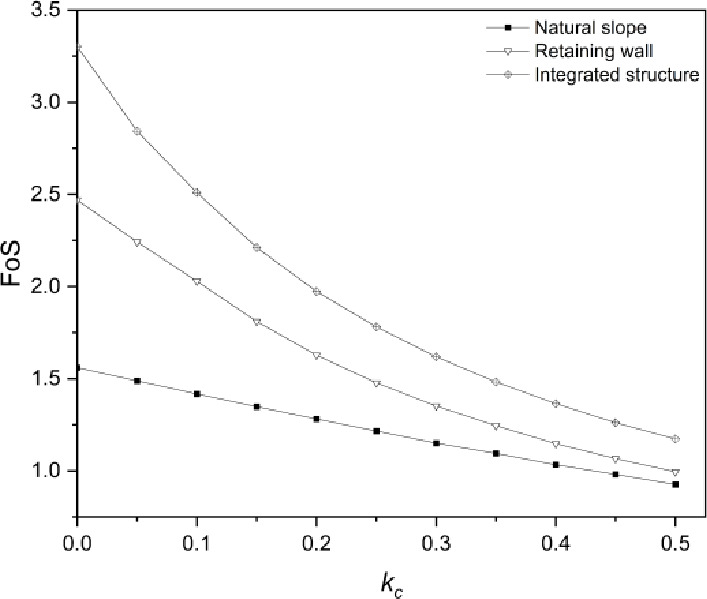
Comparison of Factor of Safety (FoS) of three slope patterns under seismic effect.

The comparison of FoS among three slope patterns under seismic effect is shown in Fig. 13. It is noticeable that the value of $\:L/H$ ratio of geogrid layers and $\:{L}_{E}/{H}_{U}$ ratio of piles is $\:1.1$ and $\:5/3$ based on actual site conditions. Under static loading conditions ($\:{k}_{c}=0$), the geogrid-reinforced soil retaining wall improves the FoS of unreinforced road cutting by $\:58.3\%$, and the FoS is doubled when unreinforced road cutting is stabilized by an integrated structure. The FoS of three slope configurations tends to decrease with the increase of $\:{k}_{c}$. When $\:{k}_{c}$ increases to $\:0.5$, the FoS of natural slope and reinforced soil retaining wall decreases significantly to around unity, which indicates a critical limit state of slopes. For the integrated structure stabilized slope under the same condition, the FoS is around $\:1.2$ which represents a relatively stable state of the slope and the improvement in FoS is estimated at $\:25\%$. It is worth noting that the effect of the integrated structure in stabilizing slopes is significant when the magnitude of the earthquake is from small to moderate, however, the slope would fail regardless of the stabilization method when the earthquake magnitude is strong.

The significant improvement in seismic capacity of the integrated structure is fundamentally derived from the synergistic working mechanism and continuous load transfer path formed by the geogrid-reinforced soil retaining wall and laterally loaded piles through steel I-beam post, which is also the core innovation of this integrated slope stabilization system. Specifically, the layered geogrids form a stable reinforced soil composite with the backfill soil through interface friction and interlocking effect, which effectively constrains the lateral deformation, and integrates the originally scattered slope soil into a continuous mass with enhanced shear strength and integrity under seismic loading. The horizontal rail is rigidly welded to the I-beam posts embedded in the concrete anti-slide piles, the lateral sliding thrust is completely transmitted to the laterally loaded piles through the I-beam support system. As piles are embedded in the stable rock layer, sufficient resisting force can be provided by the high bending stiffness of the piles and the anchoring effect, which effectively resists the overall lateral sliding of the reinforced soil retaining wall and the upper slope soil. In general, the load transfer path realizes the full coupling of the two reinforcement technologies, thus achieves a significant improvement in the seismic stability of the slope.

The pseudo-static method adopted in the current study is a widely used method for seismic stability analysis, however, it has inherent limitations. The pseudo-static approach simplifies the complex time-varying seismic dynamic load into a constant static inertial force, which ignores the time history effect of ground motion, including the influence of seismic wave duration, frequency spectrum characteristics and peak acceleration time-varying law on the seismic response of the structure. In addition, this method neglects the dynamic characteristics of the soil, such as the cumulative growth of excess pore water pressure in the soil under seismic loading, the dynamic change of soil shear modulus and damping ratio with the increase of strain, which may lead to a certain deviation between the calculated safety factor and the actual stability state of the slope under strong earthquakes. For a more refined and comprehensive analysis of the seismic capacity of this integrated structure, subsequent research will adopt dynamic time-history analysis method, input the actual seismic wave records of the South Gippsland area, and fully consider the dynamic characteristics of soil, to further improve the accuracy of the numerical simulation.

## Conclusions

In order to tackle the slope instability issue which induced by seismic effects in the South Gippsland area, an integrated slope-stabilization structure that involves laterally loaded piles and geogrid-reinforced soil retaining walls is adopted. In the current study, the response of the geogrids and the piles, and the behavior of the slope under seismic loading conditions are demonstrated, and the effect of this integrated slope-stabilization structure is also indicated. Comparing and analyzing the results from various parametric studies, it can be concluded that:

(1) For all three slope configurations, the FoS of slopes decreases with the increase of $\:{k}_{c}$. When $\:{k}_{c}$ equals $\:0$, the increase in FoS of unreinforced road cutting induced by reinforced soil retaining wall and integrated structure is $\:58.3\%$ and $\:111.7\%$, respectively. With the growth of $\:{k}_{c}$ to the maximum value adopted in this study ($\:0.5$), the percentage of increase in FoS contributed by reinforced soil retaining wall and integrated structure decreases to $\:7.5\%$ and $\:25\%$, respectively. Therefore, the effect of the integrated slope-stabilization structure on maintaining the stability of the slope and the embankment under the static loading conditions and the seismic loading conditions can be demonstrated. Although the effect of the integrated structure drops with the increase of $\:{k}_{c}$, the integrated structure can still ensure the stability of the slope configuration adopted in the current study which is based on actual site conditions.

(2) Similar to the static loading condition, there is a threshold value of geogrid embedment length under the seismic loading condition. The major improvement of FoS of geogrid reinforced soil retaining wall stabilized slope occurs when $\:L/H$ ratio ranges from $\:0.5$ to $\:1.1$. However, the effect of increasing the length of geogrid embedment on improving slope stability is minor when $\:{k}_{c}$ is larger than $\:0.25$. Regarding the laterally loaded piles which are placed at the toe of the retaining wall, the threshold value of $\:{L}_{E}/{H}_{U}$ under seismic effect ranges from $\:2/3$ to $\:5/3$, which is larger than the corresponding threshold value under static loading conditions. This phenomenon indicates that for the slope located in the seismically active area, the laterally loaded pile that adopted to improve its stability needs larger length compared with the seismically inactive area. In addition, the position of maximum pile bending moment has a tendency to move upward with the increase of $\:{k}_{c}$. For geogrid reinforced soil retaining wall stabilized slopes located in seismically active area, the optimal $\:L/H$ ratio that ranges from $\:0.5$ to $\:1.1$ is recommended. Moreover, regarding the integrated structure stabilized slopes within seismically active location, the optimal $\:{L}_{E}/{H}_{U}$ ratio ranges from $\:2/3$ to $\:5/3$. It is worth noting that these optimized design parameters are based on a specific slope site in the study area, when the slope parameter changes, these recommended design parameters can still provide a rapid and simple evaluation for the slope stabilization design.

(3) For the three slope configurations analyzed in the current study, the scale of the critical slip surface has a tendency to become larger with the increase of $\:{k}_{c}$. Moreover, this tendency changes the failure mode of the slope from horizontal sliding to global failure, and the significant decrease in FoS of slopes under large values of $\:{k}_{c}$ can be illustrated by this phenomenon. The phenomenon that the scale of the critical slip surface tends to become larger with the increase of the earthquake magnitude still needs more investigation.

The performance of three slope configurations subjected to seismic effects has been studied, and the effectiveness of the integrated structure has also been indicated clearly. As South Gippsland is one of the most seismically active areas in Australia, special attention should be paid to this issue. The pseudo-static approach is a relatively simple method that could provide a common and rapid evaluation of the seismic capability of this integrated slope-stabilization structure, the actual seismic wave should be adopted for future studies to better understand the seismic resistance of this integrated structure.

## Data Availability

Data and materials used during the study will be made available upon reasonable request to the corresponding author.

## Abbreviations

$$\:{c}^{{\prime\:}}$$: Effective cohesion
$$\:{c}_{int}^{{\prime\:}}$$: Interface cohesion
$$\:{c}_{red}$$: Reduced cohesion
$$\:{c}_{rf}$$: Reduction factor
$\:{g}_{h}$: Horizontal seismic acceleration
$$\:{g}_{v}$$: Gravitational acceleration
$\:{k}_{c}$: Critical seismic coefficient
$\:{\left(\mathrm{t}\mathrm{a}\mathrm{n}{\phi\:}^{{\prime\:}}\right)}_{red}$: Reduced friction angle
$\:{\sigma\:}_{1}$: Major principal stress
$\:{\sigma\:}_{3}$: Minor principal stress
$\:{\sigma\:}_{n}^{{\prime\:}}$: Effective normal stress
$\:{\phi\:}^{{\prime\:}}$: Effective friction angle
$\:{\phi\:}_{int}^{{\prime\:}}$: Interface friction angle
$\:{\phi\:}_{min}^{{\prime\:}}$: Minimum friction angle
